# New Insights into Adipose Tissue Metabolic Function and Dysfunction

**DOI:** 10.3390/ijms24129953

**Published:** 2023-06-09

**Authors:** Giovanni Pallio

**Affiliations:** Department of Clinical and Experimental Medicine, University of Messina, Via C. Valeria, 98125 Messina, Italy; gpallio@unime.it

Currently, one-third of people worldwide are overweight or obese, with a higher prevalence in women than in men and in the elderly than in the young. Individuals, society, and the healthcare system are all heavily affected by the obesity pandemic. Thus, overweight and obesity are independent risk factors for cardiovascular disease, which is the world’s leading cause of death and a significant source of disability, resulting in nearly five million premature deaths annually. Furthermore, visceral fat growth is closely correlated with ectopic fat deposition in skeletal muscle, heart, liver, pancreas, and blood vessels, a trend that increases pro-inflammatory cytokines levels and is associated with lipotoxicity [1,2]. Adipose tissue is an energy-processing endocrine organ that is traditionally divided by its use or anatomical distributions. White adipose tissue (WAT) (the primary lipid-storing cells) is found in nearly every portion of the human body, and it is characterized by having a low mitochondrial density and storing triglycerides in a single lipid droplet. As opposed to this, brown adipose tissue (BAT) is mostly found in the inter-scapular and mediastinal areas, and it is characterized by the presence of multilocular adipocytes, which contain many small lipid droplets and a large number of oxidative mitochondria that oxidize glucose and lipids in order to produce heat, thus dissipating energy via the process of adaptive heat production (thermogenesis). In recent years, growing evidence suggests that adipose tissue has more colors, such as beige, pink, and yellow adipocytes. Beige adipocytes appear in WAT following different stimuli: for example, cold exposure or catecholamine stimulation. These cells are the result of a white-to-brown trans-differentiation via the so-called browning process. Beige adipocytes showed some features in common with WAT and some others with BAT. Thus, these cells are characterized by a medium density of mitochondria and expressed the UCP1thermogenic marker. Furthermore, all three types of adipocytes have the ability to trans-differentiate into mammary luminal secretory epithelium cells, known as “pink adipocytes” (PAT), which are necessary for milk secretion during pregnancy and lactation. Finally, marrow adipose tissues (MAT) in bones are related to the yellow adipocytes. In healthy, lean individuals, MAT makes up more than 10% of the total fat mass. The unilocular shape of yellow adipocytes resembles that of white adipocytes, but they exhibit a unique gene expression, lipid profile, and metabolism [3]. Adipose tissue is the body’s largest endocrine organ. Thus, adipokines or batokines produced from adipose tissue not only show autocrine but also show paracrine and endocrine functions. The research articles and reviews collected in this research topic provide new insights into the pathogenesis, molecular pathways, and beneficial effects of novel and safe treatments of metabolic diseases associated with adipose tissue dysfunctions.

The review by Garcìa-Vega et al. illustrated that diabesity (a combination of type 2 diabetes and obesity) contributes to cardiac, metabolic, inflammation, and neurohumoral changes that determine cardiac dysfunction (diabesity-related cardiomyopathy). In particular, they present the mechanisms of diabesity associated with cardiovascular disease and their therapeutic implications. The authors concluded that ageing is associated with adipose tissue accumulation and insulin resistance, leading to an increase in circulating advanced glycation end products (AGEs) that enhance ROS production and reduce NO in endothelial cells, favoring atherogenesis. Lifestyles can modulate these mechanisms even for low-physical-capacity individuals during ageing, suggesting the need for new therapeutic approaches with respect to metabolic and endocrine improvements [1]. In line with these findings, the review by Ly and colleagues evidenced that obesity and, in particular, body fat distribution are closely associated with the risk of cardiovascular diseases. Moreover, the authors highlighted that the distribution of body fat differs between women and men. Thus, at similar BMI, the female fat distribution pattern is associated with improved cardiovascular risk even if ectopic fat depositions within the neck, abdomen, and pericardium are more strongly associated with women’s adverse cardiovascular risk compared to men. Finally, they reported that environmental factors may have a greater genetic impact on females than males in terms of the regulation of fat distribution and expression [2]. Additionally, Gómez-García and colleagues in their review addressed the sexual dimorphism in brown adipose tissue activation and white adipose tissue browning. They highlighted that females activate thermogenesis earlier than males when exposed to decreasing temperatures and are more sensitive than males also in the case of energy restriction. Moreover, during sexual development, an increase in BAT mass and BAT activity takes place, and this phenomenon is lower in girls than in boys, suggesting that this effect might be due to the higher muscle mass expansion in this gender. However, the opposite occurs as people age, which is a period during which thermogenic capacity decreases, and this change is more pronounced in males than in women. Finally, they reported that in the vast majority of cases, females are more prone than men to develop browning [3]. In recent years, obesity and insulin resistance were also correlated with iron overload, a condition that leads to increased inflammation, decreased insulin sensitivity, and altered adipokine release. Ameka and colleagues showed that metabolically activated bone-marrow-derived macrophages (MMe BMDMs) and adipose tissue macrophages (ATMs) from obese mice have reduced the expression of several iron-related proteins. Moreover, they found that iron exchange between BMDMs and adipocytes was regulated by macrophage phenotype. Thus, in their experimental model, MMe macrophages transferred and received more iron from adipocytes than M0, M1, and M2 macrophages. These results demonstrated that, in the MMe condition, the redistribution of iron is biased toward macrophage iron deficiency and simultaneous adipocyte iron overload, suggesting that obesity alters iron communication between adipocytes and macrophages, and correcting this iron communication channel may be a novel therapeutic target for reducing insulin resistance [4]. Moreover, Ruggiero et al. investigated the role of macrophage phenotypes in a cohort of 44 African green monkeys stratified into four clinically defined groups: (i) metabolically healthy lean (MHL), (ii) metabolically healthy obese (MHO), (iii) metabolically unhealthy obese (MUO), and (iv) metabolically unhealthy lean (MUL). They observed that the MHO group showed a significant increase in M2 macrophages and an upregulation of fatty acid oxidation related to an increase in the transcription of fatty acid binding protein 7 (FABP7). Conversely, the MUO group showed downregulation of these factors and a concomitant upregulation of immune responses. These results suggested that M2 macrophages are related to sustained metabolic health in the obese state and could be novel targets for improving metabolic health with obesity [5]. Furthermore, in two research articles, Kimura et al. and Thomalla et al. investigated the role of two receptors expressed in adipocytes: the taste 2 receptor (T2R) and the endosome-localized Toll-like receptor 7 (TLR7), respectively. In the first study, the authors showed that both fasting and stimulation by bitter compounds (well-known T2R agonists) increased the expression of *Tas2r* genes in mouse WAT. Moreover, serum starvation and stimulation by bitter compounds increased the expression of *Tas2r* genes in 3T3-L1 adipocytes, suggesting that T2Rs have a functional role in adipocytes. In particular, the overexpression of *Tas2r126* in 3T3-L1 preadipocytes decreased fat accumulation and reduced the expression of adipogenic genes, highlighting its role in adipocyte differentiation [6]. Thomalla et al analyzed the TLR7 mRNA and protein expression in several in vitro models, including 3T3-L1 cells; they observed that TLR7 expression is strongly induced during adipocyte differentiation. Moreover, they showed that the TLR7 agonist imiquimod (IMQ) treatment downregulates the expression of glucose transporter Glut4 resistin as well as the adipokine concentration in cell culture supernatants. However, considering that most data were generated in murine adipocytes, further in vivo studies are needed in order to confirm these intriguing results [7]. The other two papers published in this Special Issue investigated the role of potential biomarkers of thermogenesis activity and efficacy during obesity therapy. In this context, Camino et al. characterized the protein cargo of BAT extracellular vesicles (EVs) (i.e., batosomes) from lean and obese Sprague–Dawley rats in order to establish a reference proteome map. The authors analyzed the EVs secreted by white adipose tissue (subcutaneous and visceral WAT), showing that 60% of proteins were exclusive of BAT EVs. Thus, the batosomes of lean animals contain proteins associated with mitochondria, the electron transport chain, lipid metabolism, and the beta-oxidation pathway; on the contrary, the batosomes of obese animals showed proteins involved in signal transduction; cell communication; the immune response; inflammation; thermogenesis; and potential obesity biomarkers, including UCP1, Glut1, MIF, and ceruloplasmin. Therefore, they concluded that the protein cargo of batosomes dynamically reflects the changes in the cells of origin, carrying biomarkers of mitochondrial BAT activity and thermogenesis as well as of inflammation, and oxidative stress during the development of obesity [8]. Furthermore, Schmid and colleagues enrolled 160 severely obese patients who underwent a low-calorie diet (LCD) or Roux-en-Y gastric bypass (RYGB) and investigated the post-interventional dynamics of adipokines and hepatokines, such as ANGPTL4, CCL5, GDF15, GPNMB, and IGFBP6, as well as their correlations with fat mass reduction and the improvement in liver fibrosis. They observed that systemic GDF15 levels were an excellent predictive marker for hepatic fibrosis, type 2 diabetes mellitus, and hypertension, identifying also the specific GDF15 cut-off values for each disease. Overall, these data demonstrated that circulating adipokines and hepatokine levels, and particularly GDF15 concentrations, were correlated with glucose metabolism, liver integrity, and cardiovascular health, thus resulting in potential biomarkers for the clinical characterization of obese individuals and the success of obesity therapy strategies [9]. In light of the adipokines’ ability in modulating the production of cytokines that could affect insulin sensitivity, a paper authored by Casado et al. evaluated the effects of the chronic central administration of leptin (12 μg/day for 14 days) on the expression of key markers of lipid metabolism and its possible relationship with changes in inflammatory and insulin signaling pathways in epididymal adipose tissue. They did not observe changes in the expression of lipogenic enzymes, whereas a significant decrease in the activity of the malic enzyme and glucose-6-phosphate dehydrogenase was detected in the leptin-treated group compared to untreated animals. Furthermore, leptin administration induced a reduction in lipoprotein lipase and carnitine palmitoyl-transferase-1A expression, together with a decrease in the phosphorylation of insulin-signaling targets and a low-grade inflammatory pattern. In conclusion, they demonstrated that central leptin infusion regulated lipid metabolism, reducing epididymal fat stores and suggesting its use as a modulating agent in obesity [10]. Finally, in a very interesting paper, Di Maio and colleagues evaluated the ability of some drugs, including GW501516, sildenafil, and rosiglitazone, and Irisin, a myokine that is stimulated by muscular exercises, to induce the browning process of white adult adipocytes obtained from differentiated mesenchymal stromal cells (MSCs). Their data indicated that a pharmacological approach could be envisaged for shifting white adipocytes into brite/beige adipocytes. The irisin treatment appears as the most promising one in promoting the browning process, as demonstrated by the increased expression of UCP1, augmented mitochondrial mass, modifications in metabolism, and increased mitochondrial oxygen consumption, indicating that this promising browning agent could represent an appealing therapeutic approach for counteracting metabolic diseases and their associated obesity [11]. In conclusion, this research topic supports the relevance of macrophage phenotypes, taste 2, and Toll-like 7 receptors in the regulation of adipocyte function as well as the importance of measuring the biomarkers of metabolic and thermogenesis activity, such as batosomes, circulating adipokines, and hepatokines. Finally, the ability of some drugs and endogenous substances (i.e., leptin and irisin) to induce the browning process and regulate lipid metabolism was demonstrated. Overall, all these fascinating results suggest that new promising browning agents could represent the future of therapeutic approaches that counteract metabolic diseases and their associated obesity.

## References

[1] García-Vega D., González-Juanatey J.R., Eiras S. (2022). Diabesity in Elderly Cardiovascular Disease Patients: Mechanisms and Regulators. Int. J. Mol. Sci..

[2] Li H., Konja D., Wang L., Wang Y. (2022). Sex Differences in Adiposity and Cardiovascular Diseases. Int. J. Mol. Sci..

[3] Gómez-García I., Trepiana J., Fernández-Quintela A., Giralt M., Portillo M.P. (2022). Sexual Dimorphism in Brown Adipose Tissue Activation and White Adipose Tissue Browning. Int. J. Mol. Sci..

[4] Ameka M.K., Beavers W.N., Shaver C.M., Ware L.B., Kerchberger V.E., Schoenfelt K.Q., Sun L., Koyama T., Skaar E.P., Becker L. (2022). An Iron Refractory Phenotype in Obese Adipose Tissue Macrophages Leads to Adipocyte Iron Overload. Int. J. Mol. Sci..

[5] Ruggiero A.D., Vemuri R., Block M., DeStephanis D., Davis M., Chou J., Williams A., Brock A., Das S.K., Kavanagh K. (2022). Macrophage Phenotypes and Gene Expression Patterns Are Unique in Naturally Occurring Metabolically Healthy Obesity. Int. J. Mol. Sci..

[6] Kimura S., Tsuruma A., Kato E. (2022). Taste 2 Receptor Is Involved in Differentiation of 3T3-L1 Preadipocytes. Int. J. Mol. Sci..

[7] Thomalla M., Schmid A., Hehner J., Koehler S., Neumann E., Müller-Ladner U., Schäffler A., Karrasch T. (2022). Toll-like Receptor 7 (TLR7) Is Expressed in Adipocytes and the Pharmacological TLR7 Agonist Imiquimod and Adipocyte-Derived Cell-Free Nucleic Acids (cfDNA) Regulate Adipocyte Function. Int. J. Mol. Sci..

[8] Camino T., Lago-Baameiro N., Sueiro A., Bravo S.B., Couto I., Santos F.F., Baltar J., Casanueva F.F., Pardo M. (2022). Brown Adipose Tissue Sheds Extracellular Vesicles That Carry Potential Biomarkers of Metabolic and Thermogenesis Activity Which Are Affected by High Fat Diet Intervention. Int. J. Mol. Sci..

[9] Schmid A., Arians M., Burg-Roderfeld M., Karrasch T., Schäffler A., Roderfeld M., Roeb E. (2022). Circulating Adipokines and Hepatokines Serve as Diagnostic Markers during Obesity Therapy. Int. J. Mol. Sci..

[10] Casado M.E., Canelles S., Arilla-Ferreiro E., Frago L.M., Barrios V. (2023). Changes in Lipid Metabolism Enzymes in Rat Epididymal Fat after Chronic Central Leptin Infusion Are Related to Alterations in Inflammation and Insulin Signaling. Int. J. Mol. Sci..

[11] Di Maio G., Alessio N., Peluso G., Perrotta S., Monda M., Di Bernardo G. (2022). Molecular and Physiological Effects of Browning Agents on White Adipocytes from Bone Marrow Mesenchymal Stromal Cells. Int. J. Mol. Sci..

